# Training and capacity building in medical statistics in Sub-Saharan Africa: Impact of the London School of Hygiene & Tropical Medicine MSc in Medical Statistics, 1969 to 2021

**DOI:** 10.1002/sim.9304

**Published:** 2022-02-10

**Authors:** James R. Carpenter, Jim Todd, Kathy Baisley, John Bradley, Nazarius Mbona Tumwesigye, Patrick Musonda, Tobias Chirwa

**Affiliations:** 1 Department of Medical Statistics, London School of Hygiene & Tropical Medicine, London, UK; 2 MRC Clinical Trials Unit, UCL, London, UK; 3 TAZAMA Project, National Institute for Medical Research, Mwanza, Tanzania; 4 Department of Population Health, London School of Hygiene & Tropical Medicine, London, UK; 5 MRC International Statistics and Epidemiology Group, London School of Hygiene & Tropical Medicine, London, UK; 6 Africa Health Research Institute, Natal, South Africa,; 7 Department of Epidemiology and Biostatistics, School of Public Health, Makerere University, Kampala, Uganda; 8 Department of Epidemiology and Biostatistics, School of Public Health, University of Zambia, Lusaka, Zambia; 9 School of Public Health, University of the Witwatersrand, Johannesburg, South Africa

## 1 Introduction

A key motivation for Peter Armitage in founding the LSHTM’s MSc in medical statistics in 1969 was the recognition of an urgent need for medical statisticians—with a firm grounding in both theory and application—to collaborate with clinicians in the rapidly developing field of medical research.^1^

Inheriting its international outlook from the wider School, from the beginning the course welcomed students from all over the world, not least Sub-Saharan Africa, where there are many long-standing research collaborations including in the Gambia (where the MRC unit, https://www.mrc.gm/faq/, formerly linked to and now part of the LSHTM, traces its origins back 70+ years), Malawi (Malawi Epidemiology and Intervention Research Unit, www.meiru.info/history, established in 1980), Tanzania (eg, Grosskurth et al;^2^ Ng’weshemi et al^3^ and the establishment of the Mwanza Intervention Trials Unit in 2006, https://mitu.or.tz/) and Uganda.^4^ Reflecting this, for many years, student recruitment from Sub-Saharan Africa was rather ad-hoc, and typically linked to the needs and opportunities arising from these on-going research projects (eg, analysis of household dynamics data from Karonga, Malawi;^5^ Chirwa et al;^6^ see also Kampikaho and Tumwesigye^7^).

However, over the past 20 years pronounced demographic and economic changes in Sub-Saharan Africa (SSA) have continued apace, with corresponding shifts in both disease burden and public health priorities. The prevalence of high profile diseases such as HIV, TB and malaria remains high, but so too does the prevalence of oft neglected disease (eg, schistosomiasis, which predominantly affects the rural poor in Africa). Further, there is a growing burden of noncommunicable diseases (eg, among many, Msyamboza et al^8^), as well as the emergence of new infectious diseases (Ebola, COVID-19) and the impact of climate change.

These developments and the increasing appreciation of the importance of health research, have led many countries in SSA—despite continuing resource challenges—to expand their health research programs, collecting an increasing wealth of health data from a variety of sources (eg, among many, research cohorts at the MRC Uganda Unit, www.mrcuganda.org; the International epidemiology databases to evaluate AIDS (IeDEA);^9^ the South African Population Research Infrastructure Network SAPRIN, http://saprin.mrc.ac.za/index.html; Harling et al^10^).

Nevertheless, there remains limited analytical capacity to make full use of these data, and research often relies on statistical expertise from the North. Nontrivial challenges across the SSA region include:

- *Data:* obtaining data from routine data systems is a continuing challenge. Understaffing of health facilities or other units collecting routine data, low pay, and poor facilitation lead to poor quality of data -- meaning that considerable extra effort must be devoted by statisticians to data cleaning and resolving queries with health facilities before analyses can proceed. By contrast, though, adequately resourced sample surveys in the SSA region (such as the multicountry demographic and health surveys) produce data quality that is second to none.
  Further, while an increasing number of research institutions with well managed data are willing to have students on secondment analyse their data, nevertheless barriers remain due to the complexity of the data access processes (including obtaining ethical approval). This is a challenge for students because it eats into completion times.
- *Limited funding for statistical research:* sustained access to funding is needed to create opportunities for graduates to build their career. Currently, too many statistics graduates have no alternative but to work as program managers, carrying out only descriptive statistical analyses.
- *Suitably qualified staff*: in many institutions, there is very limited capacity to provide high quality project supervision.
- *Impact of analyses:* perhaps in part due to these issues, there remain barriers to the effective use of statistical analysis to inform policy at all levels across the region.

Alongside these challenges, the past 20 years has seen increasing recognition of the need for skilled and experienced medical statisticians in African research institutions and universities, to address health challenges, provide an evidence base to inform policy makers and health service implementation, and train the next generation of researchers. For example, Fegan et al^11^ highlights the urgent need for statisticians and data managers in Africa to effectively utilize the data revolution. In response, alumni and staff from the LSHTM, mostly alumni of the MSc in medical statistics, significantly contributed to two broad initiatives to strengthen biostatistics capacity in SSA: a UK-MRC funded fellowship program from 2003, and from 2015 the Wellcome funded Sub-Saharan Africa Consortium for Advanced Biostatistics Training.

We now describe these initiatives, before reviewing their impact and discussing the extent to which they provide useful templates for the future.

## 2 Addressing the Need

### 2.1 UK MRC funded scholarship program

The International Statistical Epidemiology Group (ISEG) at LSHTM was first established in 1972, and focuses on epidemiology and control of major public health problems, particularly infectious diseases, in low and middle income countries. With funding from the UK Medical Research Council, it has been running a successful MSc Medical Statistics Fellowship since 2003, with the goal of strengthening statistical expertise in SSA.

This 2-year Fellowship is open to applicants from all countries in the SSA region, and enables students to take the 1-year Medical Statistics MSc at LSHTM followed by a year’s placement at an African research institution with links to ISEG. Fellows receive training in standard and advanced statistical methods, including design and analysis of epidemiological studies, alongside cutting edge methods such as causal inference and models for handling missing data. During the placement, Fellows are exposed to different aspects of epidemiological research and are mentored by African statisticians and ISEG staff.

**Figure 1:**
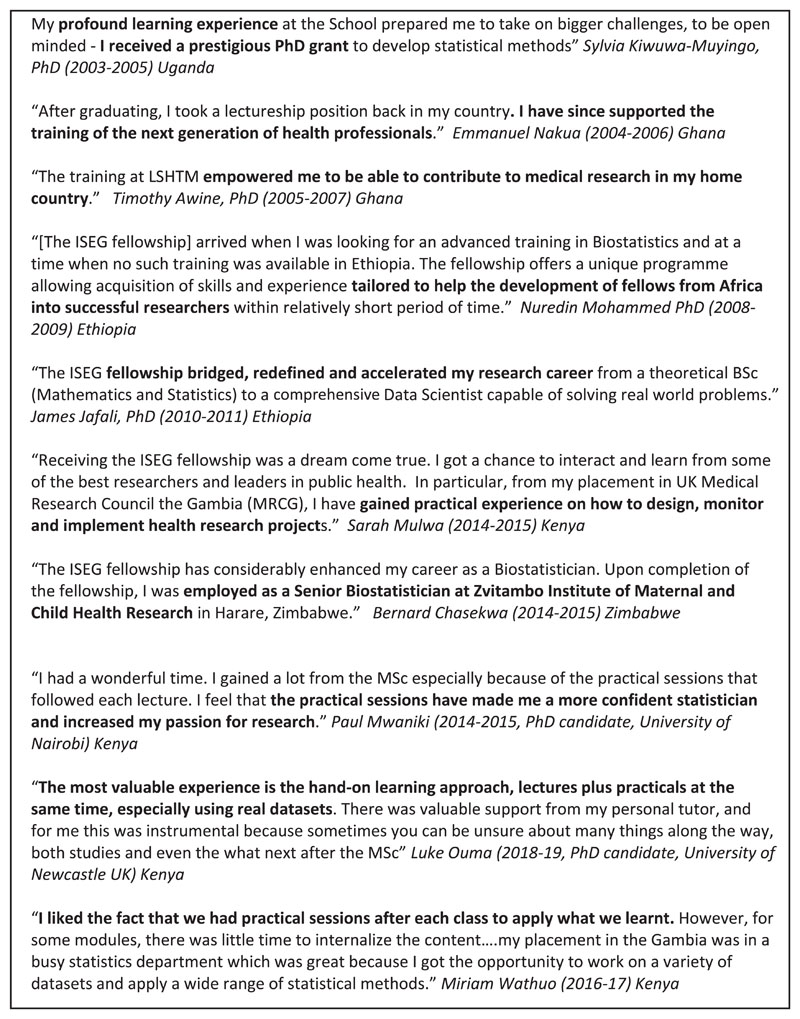
UK MRC funded Tropical Epidemiology Fellowship: fellows’ reflections on the impact of their training

To date, 25 Fellows have completed the scheme, which has achieved demonstrable impact in strengthening capacity in medical statistics in SSA. Upon enrolment, most fellows had both undergraduate degrees in mathematics and/or statistics and some experience working as statisticians in their own country (mostly managing data or producing descriptive reports). Upon graduating, most ISEG Fellows are working as medical statisticians, or studying for a PhD, in health research centers across SSA, doing both applied and methodological research. They are based in a range of institutes, including the African Population and Health Research Centre (Kenya), Ifakara Health Institute (Tanzania), Aurum Institute (South Africa); Kenya Medical Research Institute (Kenya), Zvitambo Institute of Maternal and Child Health Research (Zimbabwe), Navrongo Health Research Centre (Ghana), Kwame Nkrumah University of Science and Technology (Ghana) and the MRC LSHTM Units in The Gambia and in Uganda. In addition, six have completed a doctoral degree in biostatistics or epidemiology, two are currently studying for doctoral degrees, another will start PhD studies in 2021 and two are working in the UK. The quotes in Figure 1 reflect the impact of this fellowship.

Although the fellows’ feedback has been overwhelmingly positive, there have inevitably been challenges. One is fairly assessing the large number of candidates: putting applicants at ease and devising contextually and culturally relevant—yet appropriately searching—questions for on-line interviews is challenging. Our experience has taught us that answers to naïve questions often reveal little about ability and motivation, but more about cultural and contextual differences. To help address this, we are delighted that a former fellow Dr Nuredin Mohammed (ISEG Fellowship recipient in 2008, Assistant Professor in Medical Statistics at the MRC LSHTM Unit, The Gambia) is closely involved in shortlisting and interviewing new ISEG fellows.

Other challenges have included providing additional support to students with visa arrangements, English language test requirements (students have sometimes struggled with these formal assessments despite having good English), pastoral issues (with fellows sometimes reluctant to seek support) and the pace and nature of the assessments. Nevertheless, despite these challenges, all but one of the 25 Fellows have completed the MSc (a similar proportion to UK applicants) and while a number have required some additional tutorial support, many have performed very well, gaining merits or distinctions.

A key aspect of the Fellowship is that, after their placement, Fellows remain connected through the ISEG Fellows Network. The Network provides a framework for Fellows to engage with statistical colleagues and develop collaborations with other research groups in Africa. An exciting example is a former fellow now working in the UK successfully applying for a NIHR predoctoral fellowship for a student from Benin, who will enroll for the MSc Medical Statistics as part of their training.

### 2.2 Consortium for capacity building in Sub-Saharan Africa: https://www.ssacab.co.za/

The carefully crafted blend of theory and medical applications embodied in the modules that comprise the MSc Medical Statistics program has meant that the module materials have been used as a basis for collaborating on designing new statistics modules in Africa–both within a Masters’ program and as stand-alone short courses. For example, a co-author, Jim Todd (LSHTM, based in Tanzania) has used this model to develop modules at Kilimanjaro Christian Medical Centre, Tanzania; the University of Zambia and Kwame Nkrumah University of Science and Technology, Ghana).

These ad-hoc developments both underpinned and took more formal shape through a successful consortium bid for a Wellcome DELTAS Africa grant, led by LSHTM alumnus Prof Tobias Chirwa (Head of School at the School of Public Health University of the Witwatersrand, South Africa). With this funding, the consortium embarked on the *Sub-Saharan Africa Capacity Building in Biostatistics* (SSACAB) initiative.

The consortium aimed to plug a gap left unfilled by fellowship schemes, like the TEG described above, which can inevitably only impact a relatively small number of people. To move beyond this, high quality training in Sub-Saharan Africa needs to be nurtured, further developed and placed on a sustainable footing.

With this goal in mind, SSACAB’s first phase, from 2015 to 2020, had as its over-arching aim establishing viable masters-level statistics programs across 10 higher education centers: Kilimanjaro Christian Medical Centre, Tanzania; University of Malawi; Makerere University Uganda; University of Nairobi, Kenya; University of Zambia; University of Kinshasa, Democratic republic of the Congo; University of Namibia; University of the Witwatersrand, Stellenbosch University and University of KwaZulu-Natal in South Africa.

The consortium supported members in designing and developing the curriculum and—where this did not already exist—getting approval for a masters’ program in medical/biostatistics. Importantly, the program structure is flexible: some masters’ courses are 2years rather than one; some are taught in the “traditional” manner while others (eg, Windhoek, Namibia) use a lot of weekend teaching to allow students to combine study and work. Where local teaching was not available, consortium members were facilitated in both (a) traveling to the centers to teach masters modules, and (b) developing materials for remote learning. Crucially, the DELTAS funding supported 95 masters’ students between 2015 and 2020, providing scholarships covering fees and living costs for students to be trained on masters’ programs across the partner institutions. This provided substantial support to the partner institutions masters’ programs. Student recruitment focussed on establishing basic statistical competence and potential, and included a number of clinicians.

Central to the consortium’s vision is to demonstrate that internationally excellent education in medical statistics can be obtained in the region. To this end, SSACAB is sponsoring accreditation of partner institutions masters’ programs by the Royal Statistical Society (UK). To date, the University of Witwatersrand has been accredited, and the accreditation process continues with Stellenbosch University, the University of KwaZulu-Natal, the University of Malawi and Kilimanjaro Christian Medical Centre.

The LSTHM’s Medical Statistics MSc underpinned this work in three ways:

Firstly, not only was LSHTM a Northern Partner in SSACAB (with five staff members supporting, developing and teaching on the new masters programs), but also many of the personnel in the consortium taught and/or studied on the Med Stats course in LSHTM. The shared experience of these alumni and staff helped define the way that Masters programs in statistics, medical statistics and epidemiology and applied biostatistics were developed—preserving a carefully constructed blend of theory and medical applications, with a strong emphasis on developing those practical statistical skills essential for a statistician to work as part of a health research project team.

Secondly, much of the curriculum developed by the SSACAB partners for the new programs drew heavily on the structure of the LSHTM medical statistics program. This borrowing included both material for the foundation courses which provide the basis for statistical expertise, and optional modules available to students for advanced learning. The structures of the SSACAB programs are described in more detail by Chirwa et al.^12^

Thirdly the content and approach to biostatistics teaching has been heavily influenced by the LSHTM medical statistics program, a key component of which is complementing each formal lecture with a practical session which is at least as long, where students analyse data using the techniques they have just learned. In these practical sessions, systematic emphasis is also placed on training statisticians to effectively relate their analyses to the medical context, and report their results in a structured, accessible format—so training them to be effective members of broad-based research teams. These emphases were not common in many SSA statistics programs, and were important to cultivate given they have been greatly valued by the ISEG fellows (Figure 1).

These aspects mean that the SSACAB Masters’ programs developed to effectively equip students both to work effectively as members of a research team and to initiate research. This continues to be supported by provision of scientific writing courses at SSACAB sponsored regional conferences. Together, this ensures SSACAB graduates have the skills needed to work effectively as statisticians in health research teams—not least skills in designing and analysing intervention and observational studies.

Students’ have embraced the opportunities provided by SSACAB with sustained commitment and enthusiasm. Their progress and work have been impressive, witnessed by 54 current publications, including in *Lancet Global Health* (Abuga et al);^13^ *Epidemiology* (Yende-Zuma et al)^14^ and *Statistical Methods in Medical Research* (Gachau et al).^15^ We are particularly delighted that two female SSACAB graduates have now completed PhDs.

Looking forward, we are delighted that a second, 4 year, phase of SSACAB has been funded, with an increased number of Francophone partners and an emphasis on supporting PhDs and postdoctoral researchers.

## 3 Discussion

Above we have highlighted how the LSHTM medical statistics program has strongly influenced, and continues to support and foster biostatistics capacity building in SSA. This has been a reciprocal process, however, and much has been learned from SSA students and colleagues, in particular (i) practical approaches for addressing cultural issues; (ii) approaches to developing students whose prior education has had a strong focus on factual recall, and (iii) how to encourage an educational culture in which students engage with each other and are not excessively deferential to staff.

Alongside these schemes, there are many other similar initiatives. These include (i) the Stellenbosh based Consortium for Biostatistical Excellence in Sub-Saharan Africa;^15^ the African Institute for Mathematical Sciences https://nexteinstein.org/); (iii) the Moi-Brown Parnership for HIV biostatistics training, (which was awarded $1.6 M further funding in May 2021) and (iv) the interdisciplinary malaria research training in Malawi (which has a strong biostatistics component). But how effective are these types of schemes, and where should future efforts focus?

Both the ISEG fellowships, SSACAB, and other similar schemes, have increased the number of skilled biostatisticians available to support medical researchers in SSA, with strong interdisciplinary skills geared toward local research needs. These schemes have provided a foundation for medical statisticians to undertake advanced PhD training, further developing their skills and expertise to become research leaders in SSA. In this way, they have contributed to the building of an internationally competitive, sustainable network of medical statisticians across SSA, strengthening the capacity for African-led research. However, the ISEG fellowships are—by their nature—highly selective, and hence the focus is on training future research leaders. By contrast the—admittedly much more substantial—investment in SSACAB has made tangible progress toward the goal of establishing and nurturing internationally accredited medical statistics programs across SSA, which will train not only future research leaders, but a broad community of statisticians for health research.

Complementing educational programs, we believe that establishing effective professional biostatistics networks across SSA is a key element in fostering research culture. To this end SSACAB has linked with the International Biometric Society, through its Sub-Saharan Africa Network (SUSAN), with many SSACAB students and ISEG fellows presenting their work at regional conferences in Malawi and South Africa. To the same end, SSACAB was delighted to secure funding for a 4-year second phase, with greater emphasis on PhD and postdoctoral training.

What lessons can be learned from SSACAB? The fact that many of the co-applicants had previously worked together meant that SSACAB gelled well and quickly, even though on paper it appears to consist of a disparate set of training and research institutions. Further, the role of the northern partners in both providing teaching and supporting others’ teaching enabled provision of an up-to-date core curriculum to internationally recognized standards, as well as supporting PhD supervision. This provides a good model for future collaborations. Further, the need for consortia such as SSACAB to include resources for strengthening institutional support for research clearly emerged: the capacity for financial and administrative management varies greatly between institutions (and issues typically only emerge once funding is awarded). This aspect is crucial to retain the confidence of funders, and hence for delivering sustainable change.

Looking forward, the COVID-19 pandemic has accelerated the move toward a mix of face-to-face and on-line learning, and this model holds considerable promise for providing specialist modules for masters’ programs across SSA. However, on the negative side, the UK’s drastic cuts to Official Development Assistance (ODA) funding are a direct threat to UK institutions’ research collaborations in SSA. For example, the MRC/DFID/FDCO’s Africa Research Leadership scheme has been canceled. More generally, the funding uncertainties for ongoing and planned projects may negatively impact on training initiatives and capacity strengthening programs.

Perhaps the most encouraging aspect of all the work discussed here is that it is a genuine partnership between all those involved. And its ultimate success? We can see the first fruits already, with ISEG and SABBAC alumni contributing to African-led research and African-led capacity building. Continued networking suggests that much more fruit is ripening, and this will increase the proportion of locally-led, locally-owned biostatistical research in SSA, effectively responding to local priorities. We are working to connect with national governments and companies, since their support and resources are crucial, in the longer term, to developing and sustaining attractive careers in statistical innovation and application. We believe the goal of an internationally recognized flourishing SSA statistical community is within the reach of the next generation of researchers, and alumni and staff of the LSHTM’s Medical Statistics Program are delighted to partner with others in working enthusiastically to this end.

## Data Availability

Data sharing is not applicable to this article as no new data were created or analyzed in this study.
